# Interpretation of myocardial FDG uptake during corticosteroid therapy in cardiac sarcoidosis evaluation with ^18^F-FDG PET/CT

**DOI:** 10.1007/s12149-026-02222-z

**Published:** 2026-05-11

**Authors:** Osamu Manabe, Shiro Watanabe, Kenji Hirata, Tadao Aikawa, Takahiro Sato, Toshiyuki Nagai, Keiichi Magota, Ichizo Tsujino, Noriko Oyama-Manabe

**Affiliations:** 1 Department of Radiology, Jichi Medical University Saitama Medical Center, 1-847 Amanuma-cho, Omiya-ku, Saitama, 330-8503 Japan; 2 Department of Nuclear Medicine, Hokkaido University Graduate School of Medicine, Sapporo, Japan; 3 Department of Cardiovascular Biology and Medicine, Juntendo University Graduate School of Medicine, 2-1-1 Hongo, Bunkyo-ku, Tokyo, 113-8421 Japan; 4 First Department of Medicine, Hokkaido University Hospital, Sapporo, Japan; 5 Department of Cardiovascular Medicine, Faculty of Medicine, Graduate School of Medicine, Hokkaido University, Sapporo, Japan

**Keywords:** Cardiac sarcoidosis, Positron emission tomography, Fluorodeoxyglucose, Corticosteroid therapy, Physiological uptake

## Abstract

**Purpose:**

Cardiac sarcoidosis (CS) is a potentially life-threatening condition, and ^18^F-fluorodeoxyglucose (FDG) positron emission tomography (PET) is highly sensitive for detecting myocardial inflammation. However, corticosteroid therapy may alter myocardial metabolism, complicating the interpretation of FDG PET. This study aimed to evaluate the impact of corticosteroid therapy on physiological myocardial FDG uptake and assess the utility of fasting plasma glucose (FPG) and free fatty acids (FFA) in distinguishing physiological from pathological uptake.

**Materials and methods:**

We retrospectively analyzed FDG PET/CT scans of 40 CS patients who underwent paired scans before and after corticosteroid therapy, following prolonged fasting (> 18 h). The frequency of physiological myocardial FDG uptake was compared, and FPG and FFA levels were assessed as predictors using receiver operating characteristic (ROC) analysis. Exploratory analyses were also performed to assess metabolic changes across different levels of corticosteroid exposure.

**Results:**

Physiological myocardial FDG uptake significantly increased after corticosteroid therapy (from 2.5% to 15%, *P* = 0.048). Post-treatment FFA levels were significantly lower, while FPG levels were higher. An FFA cutoff of 615 µEq/L predicted physiological uptake with 100% sensitivity and 83.6% specificity (AUC = 0.96, *P* < 0.001), whereas FPG showed poor discrimination (AUC = 0.43, *P* = 0.83). Patients with physiological uptake had significantly lower FFA levels; FPG did not differ between groups. In analyses stratified by corticosteroid dose, physiological uptake was less frequently observed at lower dose levels, accompanied by relatively preserved metabolic parameters.

**Conclusion:**

Corticosteroid therapy increases physiological myocardial FDG uptake in CS patients, likely due to reduced FFA levels and altered metabolism. FFA may serve as a useful marker for distinguishing physiological FDG uptake from abnormal uptake, supporting more accurate PET interpretation during corticosteroid therapy.

**Supplementary Information:**

The online version contains supplementary material available at 10.1007/s12149-026-02222-z.

## Introduction

 Sarcoidosis is a chronic, multisystem granulomatous disorder of unknown etiology, characterized by the formation of non-caseating granulomas in various organs, including the lungs, skin, eyes, and heart. Although the exact cause remains unclear, immune dysregulation is considered a central factor in pathogenesis, with genetic, environmental, and infectious factors potentially contributing to its onset. The clinical course of sarcoidosis is highly variable, ranging from asymptomatic cases that resolve spontaneously to severe, chronic, and progressive disease that leads to significant organ dysfunction [1]. Cardiac sarcoidosis (CS) is a potentially life-threatening manifestation that affects the myocardium. Granulomatous inflammation in the heart can lead to structural and functional changes, including myocardial fibrosis. These alterations can result in conduction system abnormalities such as complete heart block, ventricular arrhythmias, and heart failure. These complications may progress to sudden cardiac death, highlighting the critical need for early and accurate diagnosis to prevent severe cardiac events [2].

^18^F-fluorodeoxyglucose (FDG) positron emission tomography (PET) has emerged as a crucial imaging modality for evaluating CS. FDG PET offers high sensitivity for detecting active inflammation and can assess response to therapy and early recurrence [3, 4]. FDG is a glucose analog that accumulates in metabolically active tissues, enabling visualization of inflammatory lesions. However, the myocardium utilizes both free fatty acids (FFA) and glucose as primary energy sources. In certain physiological conditions, such as the postprandial state, myocardial glucose uptake can increase, leading to physiological FDG uptake that may obscure inflammatory accumulation. This can reduce the specificity of FDG PET for detecting CS. To enhance diagnostic accuracy, pre-scan preparation involving prolonged fasting and a low-carbohydrate diet (LCD) is recommended, as these measures elevate plasma FFA levels, promoting fatty acid oxidation and minimizing physiological FDG uptake [5, 6].

Corticosteroids are the primary treatment for CS due to their immunosuppressive and anti-inflammatory properties. Moderate- to high-dose corticosteroid therapy is commonly initiated in patients with active disease to prevent irreversible cardiac damage [7, 8]. However, corticosteroids significantly impact systemic metabolism, leading to increased insulin resistance, gluconeogenesis, and altered lipid metabolism [9]. These metabolic effects can result in elevated fasting plasma glucose (FPG) and reduced FFA levels, potentially impairing the suppression of physiological FDG uptake, even when standard pre-scan preparation protocols are followed.

While FDG PET is widely used to monitor treatment response in CS, the influence of corticosteroid-induced metabolic changes on physiological myocardial FDG uptake remains poorly understood. Inadequate suppression of physiological uptake could lead to false-positive findings, misinterpretation of disease activity, and inappropriate therapeutic decisions. To date, no studies have specifically examined the impact of corticosteroid therapy on myocardial substrate metabolism and its effect on FDG uptake in the evaluation of CS.

This study aims to evaluate the effects of corticosteroid therapy on the frequency of physiological myocardial FDG uptake in patients with cardiac sarcoidosis. Additionally, we seek to assess the changes in fasting plasma FFA and FPG levels before and after corticosteroid therapy and explore their association with physiological myocardial FDG uptake, with particular attention to potential dose-dependent effects of corticosteroids on myocardial metabolism and FDG uptake.

## Materials and methods

### Subjects

This study was a retrospective evaluation of clinical records from patients diagnosed with CS between January 2009 and May 2017. The study was approved by the Institutional Review Board, with a waiver of the requirement for written informed consent.

### Study design

**Fig. 1:**
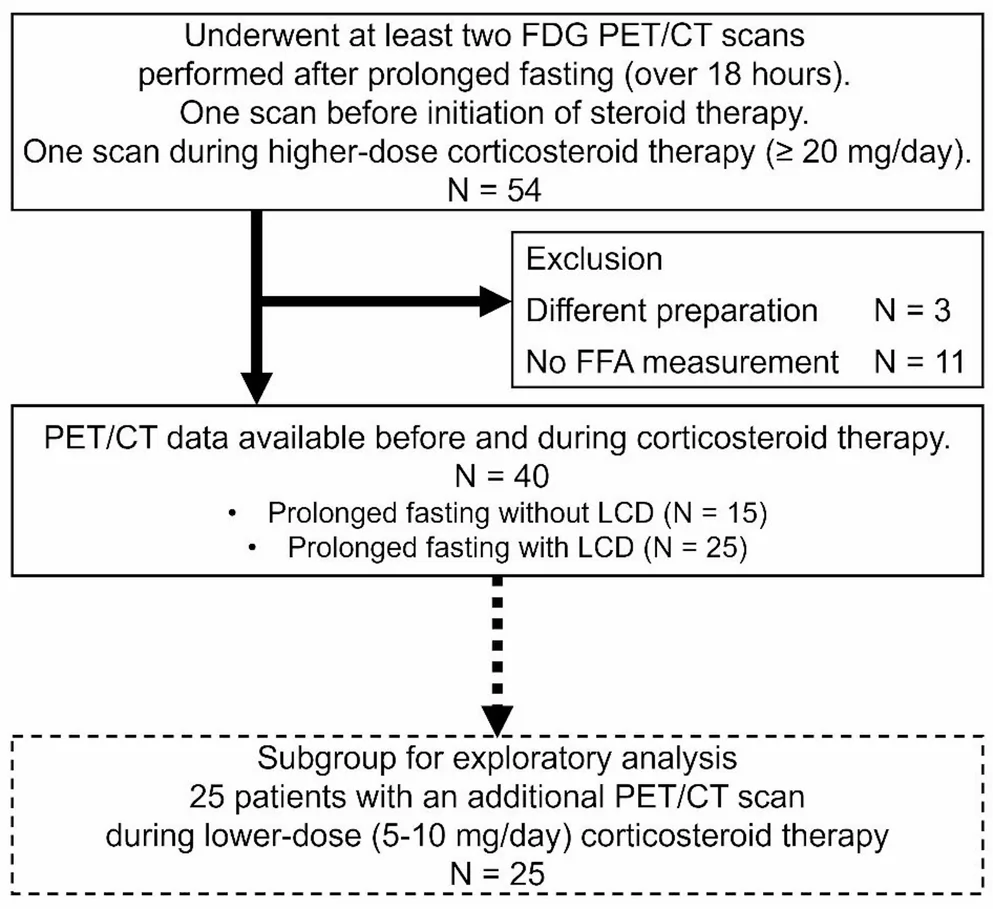
Flowchart of patient selection and subgroup classification. A total of 54 patients with cardiac sarcoidosis who underwent at least two FDG PET/CT scans after prolonged fasting (> 18 h) were initially identified. Each patient underwent one scan before initiation of corticosteroid therapy and one during higher-dose corticosteroid therapy (≥ 20 mg/day). Among these, 3 patients were excluded due to differences in preparation protocols and 11 due to missing free fatty acid (FFA) data. The final study population consisted of 40 patients with paired PET/CT data obtained before and during corticosteroid therapy. These patients were further classified according to dietary preparation: 25 underwent prolonged fasting with a low-carbohydrate diet (LCD), and 15 underwent prolonged fasting without LCD. A subgroup of 25 patients underwent an additional FDG PET/CT scan during lower-dose corticosteroid therapy (5–10 mg/day), enabling exploratory analysis across three treatment phases

The inclusion criteria for the study were as follows: [1] patients diagnosed with CS based on the according to the Japanese Circulation Society (JCS) guidelines [10]; [2] those who underwent at least two FDG PET/CT scans (i.e., before and after corticosteroid therapy) with fasting for more than 18 h before the scans; and [3] those who were imaged during the post-treatment period with a dosage of corticosteroid exceeding 20 mg/day. Patients were excluded if they did not follow the same preparation protocol for both scans or if FFA measurements were unavailable. In a subset of patients, an additional follow-up FDG PET/CT scan was available during ongoing corticosteroid therapy at lower doses (approximately 5–10 mg/day). These cases were included in an exploratory analysis to assess potential dose-dependent changes in myocardial FDG uptake patterns (Fig. 1).

We compared FDG PET/CT scans obtained before (pre-treatment) and after (post-treatment) the initiation of corticosteroid therapy. This study primarily aimed to evaluate the frequency of physiological myocardial FDG uptake before and after corticosteroid therapy. Additionally, we investigated the relationship between FFA and fasting plasma glucose (FPG) levels and the presence of physiological myocardial FDG uptake. The diagnostic performance of FFA and FPG levels in predicting physiological uptake was also assessed.

When following the LCD protocol, the meal consisted of less than 5 g of carbohydrates, including a boiled egg, tofu with bonito flakes, and grilled chicken breast with stir-fried vegetables, consumed the night before the scans [6].

### PET/CT acquisition protocol

PET/CT imaging was performed using a Biograph 64 TruePoint with TrueV (Siemens Japan, Tokyo). A low-dose CT scan was acquired for attenuation correction. Sixty minutes after FDG administration, a static FDG PET scan was performed. All scans were conducted using whole-body imaging. Blood samples were collected before FDG injection to measure FPG and FFA levels. Approximately 4.5 MBq/kg of FDG was intravenously administered under resting conditions.

### PET/CT imaging analysis

Two board-certified nuclear medicine physicians (OM and KH) independently reviewed all FDG PET/CT images in a randomized order, blinded to clinical information, treatment status, laboratory data, and each other’s interpretations. Myocardial FDG uptake patterns were initially classified into five predefined qualitative patterns (none, diffuse, focal, focal-on-diffuse, and isolated basal or lateral uptake) based on previously reported approaches [11]. Representative FDG PET images for each uptake category are provided in Supplementary Figure. For the purpose of clinical interpretation, these patterns were further categorized into two groups: physiologic and non-physiologic uptake. Diffuse homogeneous uptake or basal/papillary muscle–predominant uptake without discrete focal accentuation was considered physiologic. Focal uptake clearly exceeding adjacent myocardial background, with or without diffuse uptake, was considered non-physiologic. In cases with mixed or borderline patterns, classification was based on whether a discrete focal component could be identified. Discrepancies were resolved by consensus reading. During consensus interpretation, available multimodality imaging and, when applicable, prior imaging studies were reviewed as supportive information.

### Statistical analysis

Continuous variables are presented as medians with interquartile ranges (IQRs), while categorical variables are expressed as absolute numbers with percentages. The Wilcoxon signed-rank test was used to analyze continuous variables, and the chi-square test was employed for comparisons of categorical data. Receiver operating characteristic (ROC) analysis was performed to evaluate whether physiological myocardial uptake could be distinguished based on FFA or FPG levels using data from all PET/CT examinations, including both pre-treatment and post-treatment scans. A P-value < 0.05 was considered statistically significant. All statistical analyses were performed using JMP version 17 (SAS Institute, Cary, NC).

## Results

### Characteristics of CS patients

We included patients with CS who underwent at least two FDG PET/CT scans following a prolonged fasting period (over 18 h) before and after the corticosteroid therapy (*n* = 54). Patients were excluded if they did not follow the same preparation protocol for both scans (*n* = 3) or if FFA measurements were unavailable (*n* = 11). The final study population comprised 40 patients (9 men and 31 women), aged 27 to 75 years (median: 64 years, IQR: 51.8–68.8) (Table 1). Of the patients, 25 (62.5%) followed a low-carbohydrate diet (LCD) protocol (Protocol 1), while the remaining 15 (37.5%) underwent the protocol without LCD (Protocol 2) (Fig. 1). All patients had histologically confirmed extra-cardiac sarcoidosis and were diagnosed with CS based on the according to the JCS guidelines.

**Table 1:** Participant’s characteristics

Characteristic	Overall (*n* = 40)
Age (years)	64.0 (51.8–68.8)
Male, n (%)	9 (20.5)
Diabetes mellitus, n (%)	5 (12.5)
BMI (kg/m^2^)	21.5 (19.8–25.0)
During 1st and 2nd PET (days)	49.5 (42–70)

BMI; body mass index, PET; positron emission tomography

Continuous variables are presented as median (interquartile range). Categorical variables are presented as number of patients (%)

A subset of patients (*n* = 25) underwent an additional FDG PET/CT scan during lower-dose corticosteroid therapy and were included in a separate exploratory analysis

### Frequency of physiological uptake

**Fig. 2:**
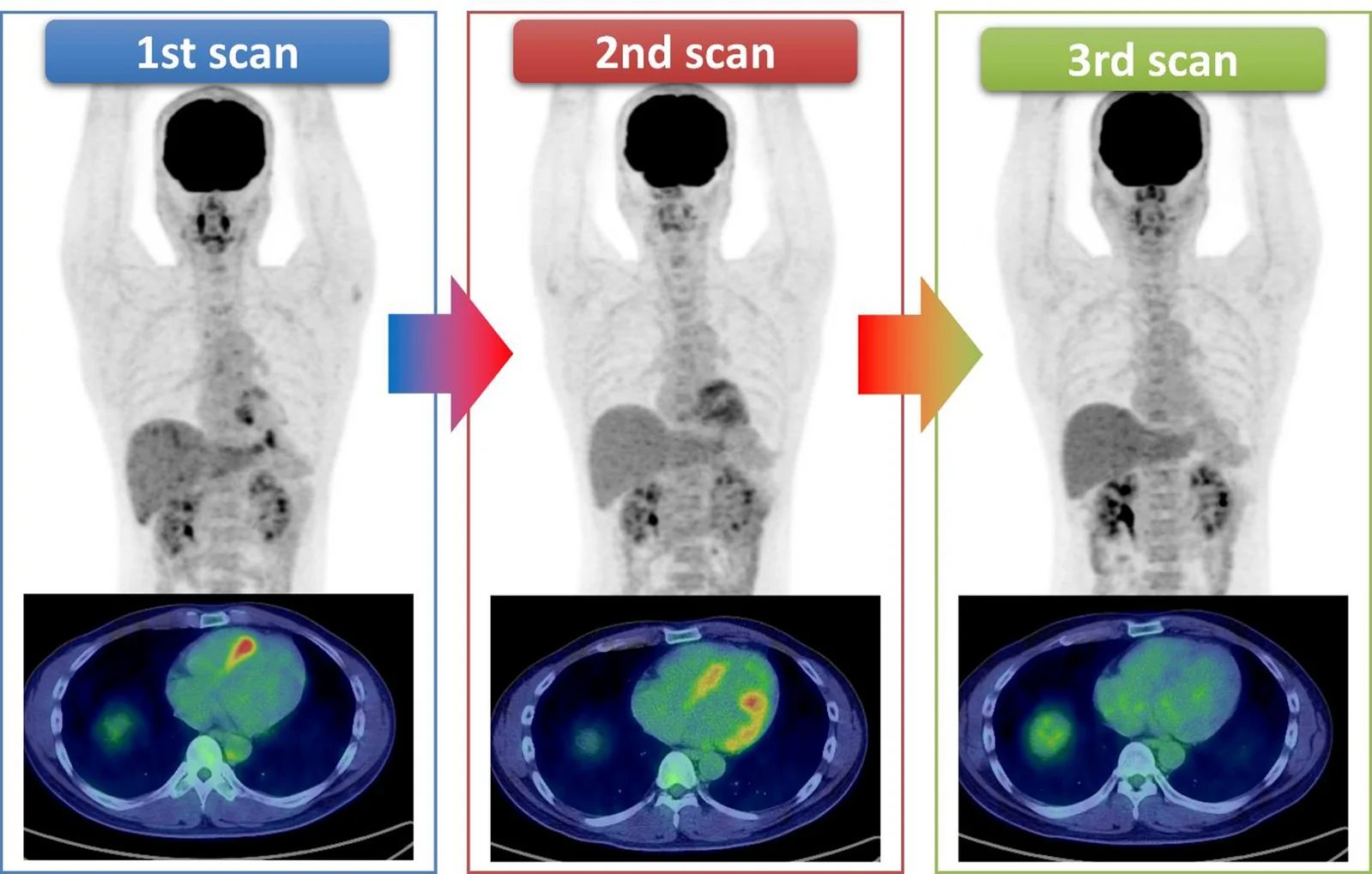
Serial changes in myocardial FDG uptake patterns in a representative patient with cardiac sarcoidosis. Whole-body maximum intensity projection (MIP) images (upper row) and corresponding axial fused PET/CT images (lower row) are shown. Before corticosteroid therapy (left), focal myocardial FDG uptake is observed. After initiation of prednisolone (PSL) at 30 mg/day (middle), the uptake pattern changes to predominantly basal/lateral uptake without diffuse background activity. During follow-up at a reduced dose of PSL (10 mg/day) (right), myocardial FDG uptake is completely suppressed. These temporal changes support the interpretation that the post-treatment uptake represents physiological myocardial FDG uptake rather than residual inflammatory activity

In the consensus reading, physiological myocardial uptake was observed in one patient (2.5%) during the pre-treatment scan and in six patients (15.0%) during the post-treatment scan. Physiological myocardial uptake was significantly more frequent during the post-treatment scan compared to the pre-treatment scan (*P* = 0.048) (Fig. 2).

### FPG and FFA levels

**Fig. 3:**
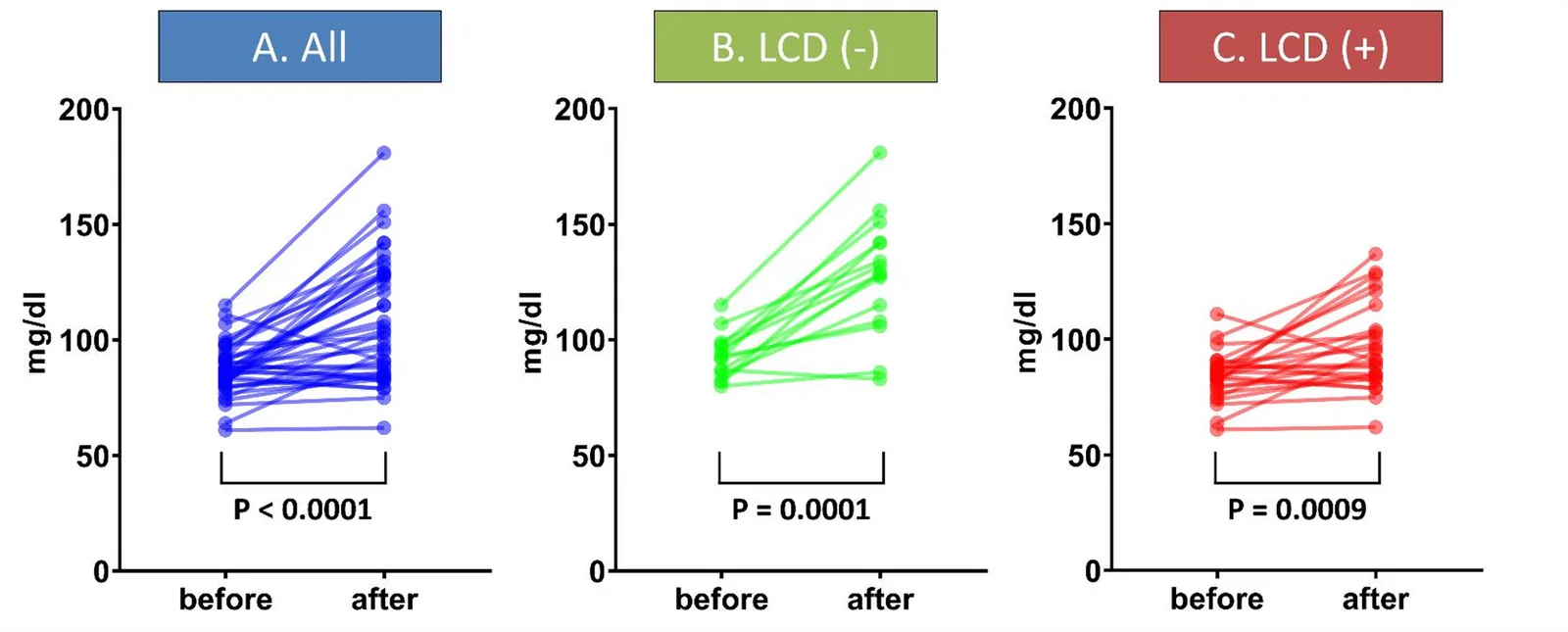
Changes in fasting plasma glucose (FPG) levels before and after corticosteroid therapy. Paired FPG levels measured before and after the initiation of corticosteroid therapy are shown for all patients (**A**), patients who underwent prolonged fasting without a low-carbohydrate diet [LCD (–)] (**B**), and patients who underwent prolonged fasting with a low-carbohydrate diet [LCD (+)] (**C**). FPG levels significantly increased after steroid therapy in all groups. Each line represents an individual patient

**Fig. 4:**
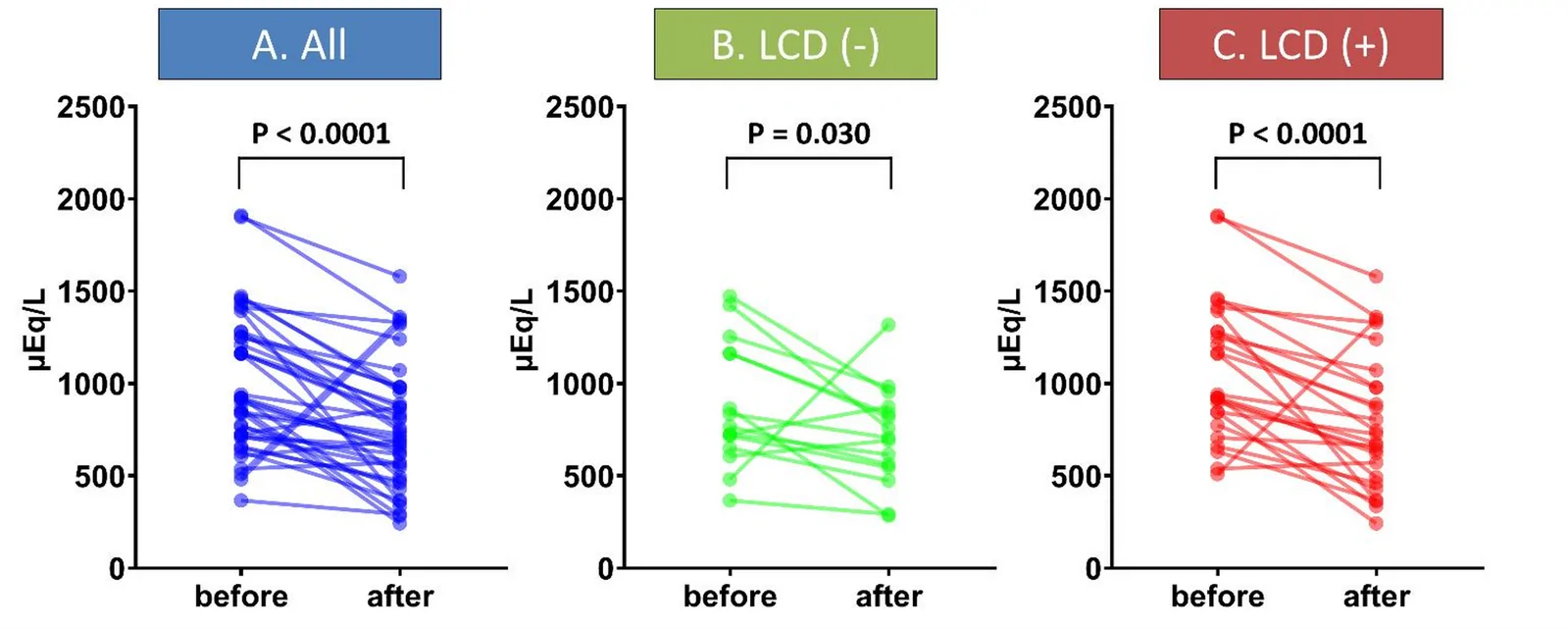
Changes in fasting plasma free fatty acid (FFA) levels before and after steroid therapy. Paired FFA levels measured before and after the initiation of corticosteroid therapy are shown for all patients (A), patients who underwent prolonged fasting without a low-carbohydrate diet [LCD (–)] (B), and patients who underwent prolonged fasting with a low-carbohydrate diet [LCD (+)] (C). In all groups, FFA levels significantly decreased after steroid therapy. Each line represents an individual patient

The values of FPG and FFA prior to FDG administration for each PET/CT examination are shown in Table 2. Both FPG and FFA levels showed significant differences between the pre-treatment and post-treatment scans, regardless of whether an LCD preparation was used. At the post-treatment scan, FPG levels were significantly higher (Fig. 3) and FFA levels were significantly lower (Fig. 4) compared to the pre-treatment scan (Table 3).

**Table 2:** Comparison of metabolic and preparation parameters

	Preparation	1st scan	2nd scan	P value
FPG (mg/dL)	All (*n* = 40)	86.5 (81.3–92.8)	103.5 (85.3-128.8)	< 0.0001
	LCD (+) (*n* = 25)	84.0 (78.0–89.0)	91.0 (83.5-109.5)	0.0009
	LCD (-) (*n* = 15)	92.0 (85.0–98.0)	129.0 (108.0-142.0)	0.0001
FFA (µEq/L)	All (*n* = 40)	920.0 (718.0-1273.5)	716.5 (506.3-971.3)	< 0.0001
	LCD (+) (*n* = 25)	940.0 (809.0-1336.5)	727.0 (477.5–1026.0)	0.0002
	LCD (-) (*n* = 15)	767.0 (650.0-1163.0)	706.0 (546.0-874.0)	0.030
Fasting period (hours)	All (*n* = 40)	20.6 (19.8–21.5)	20.5 (20.0-21.4)	0.69
	LCD (+) (*n* = 25)	20.6 (20.0-21.6)	20.5 (20.0-21.2)	0.93
	LCD (-) (*n* = 15)	20.5 (19.6–21.5)	21.0 (19.7–21.4)	0.66

FPG, fasting plasma glucose; FFA, free fatty acid; LCD, low-carbohydrate diet

Continuous variables are presented as median (interquartile range)

**Table 3:** Exploratory analysis was performed in a subset of patients with serial imaging across three time points (*n* = 25)

Variable	Pre-treatment	Higher corticosteroid exposure (≥ 20 mg/day)	Lower corticosteroid exposure (5–10 mg/day)	Comparison	*P* value
FFA (µEq/L)	864 (726–1247)	692 (493–977)	857 (676–1111)	Pre vs. Higher	0.0018
				Higher vs. Lower	0.075
				Pre vs. Lower	0.747
FPG (mg/dL)	88 (82–98)	96 (85–132)	87 (77–102)	Pre vs. Higher	0.0014
				Higher vs. Lower	0.0019
				Pre vs. Lower	0.943

FPG, fasting plasma glucose; FFA, free fatty acid; LCD, low-carbohydrate diet

Continuous variables are presented as median (interquartile range)

### Association between FFA/FPG levels and physiological myocardial uptake

**Fig. 5:**
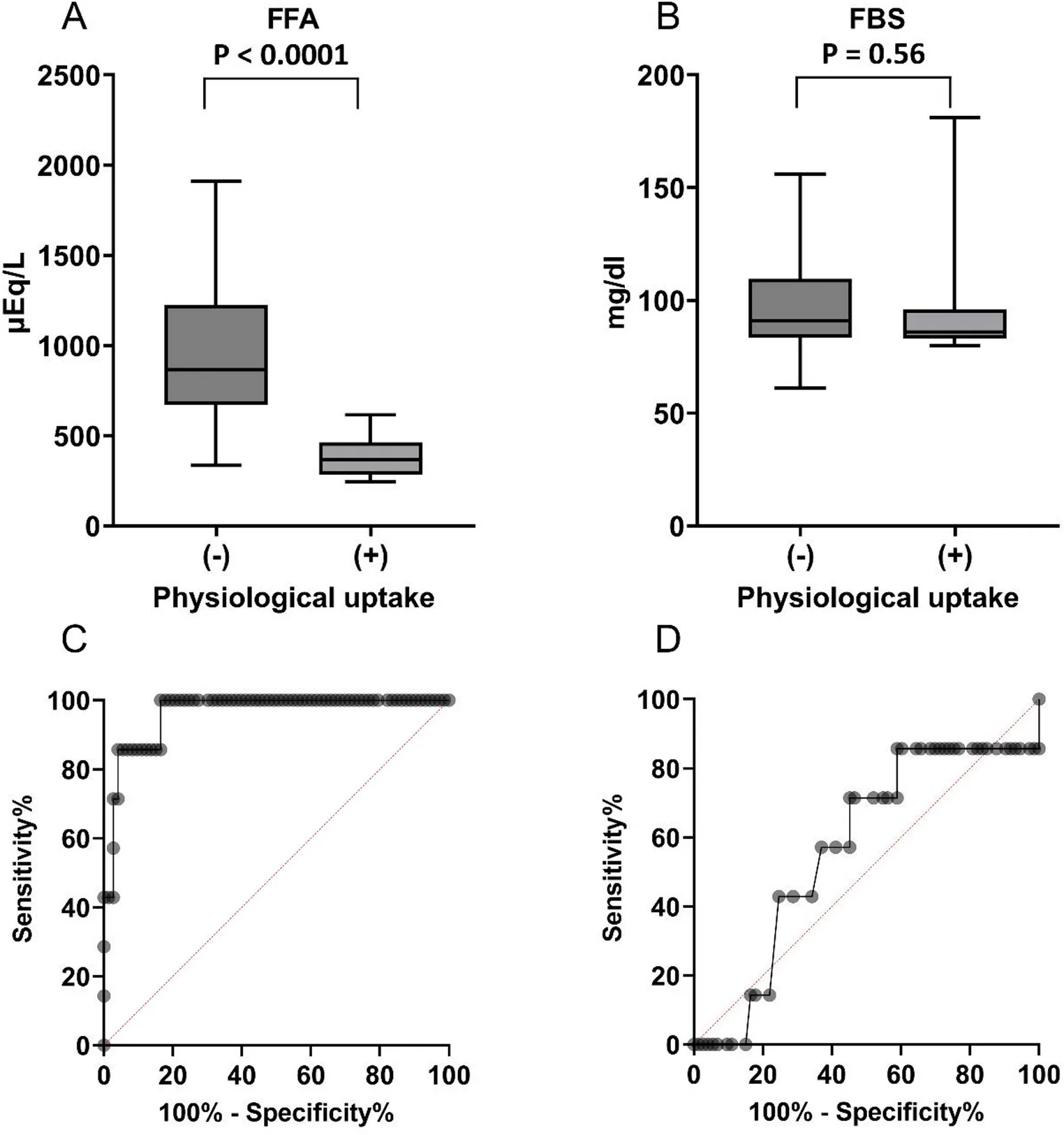
Discriminatory performance of plasma FFA and FPG levels for predicting physiological myocardial FDG uptake. Panels A and B show box plots comparing biomarker levels between individuals with and without physiological myocardial FDG uptake. Plasma free fatty acid (FFA) levels (A) were significantly lower in those with physiological uptake, whereas fasting plasma glucose (FPG) levels (B) did not differ significantly between the two groups. Panels C and D display receiver operating characteristic (ROC) curves assessing the ability of these biomarkers to discriminate physiological uptake. FFA levels (C) demonstrated excellent discriminatory performance, with an area under the curve (AUC) of 0.96 (*P* < 0.001); a cutoff of 615 µEq/L yielded 100% sensitivity and 83.6% specificity. In contrast, FPG levels (D) showed poor performance, with an AUC of 0.43 (*P* = 0.83); although the cutoff of 80 mg/dL achieved 100% sensitivity, specificity was only 15.1%. These analyses were performed using paired pre- and post-treatment data; follow-up (third scan) data were not included, as biomarker levels were not consistently available at that time point and were therefore excluded to ensure analytical consistency

When paired pre- and post-treatment scans from 40 individuals (total of 80 scans) were analyzed, ROC analysis demonstrated that an FFA cutoff value of 615 µEq/L could distinguish the presence of physiological myocardial uptake with a sensitivity of 100%, specificity of 83.6%, and an area under the curve (AUC) of 0.96 (Fig. 5A). In contrast, using an FPG cutoff value of 80 mg/dL yielded a sensitivity of 100% but a low specificity of 15.0%, with an AUC of 0.43, indicating poor discriminatory performance (Fig. 5B).

When comparing patients with physiological myocardial uptake (FFA: median 367 µEq/L, IQR: 283–462) and those without (FFA: median 864 µEq/L, IQR: 671–1225), the group with physiological uptake had significantly lower FFA levels (*P* < 0.001) (Fig. 5C). In contrast, FPG levels did not differ significantly between patients with physiological uptake (median: 86 mg/dL, IQR: 83–96) and those without (median: 91 mg/dL, IQR: 83.5–109.5; *P* = 0.56) (Fig. 5D).

### Exploratory analysis of maintenance-phase corticosteroid therapy

In a subset of patients with available serial imaging at three time points (pre-treatment, post-initiation, and lower-dose corticosteroid therapy; *n* = 25), additional analyses were performed to evaluate metabolic and imaging changes across different treatment phases. Plasma FFA levels were significantly lower during higher corticosteroid exposure compared with pre-treatment, whereas during lower-dose corticosteroid therapy, FFA levels were not significantly different from pre-treatment values. Similarly, FPG levels were elevated during higher corticosteroid exposure but returned to levels comparable to pre-treatment during lower-dose therapy. Physiological myocardial FDG uptake was infrequent during lower-dose corticosteroid therapy (1/25, 4.0%), compared with higher frequency observed during higher corticosteroid exposure.

## Discussion

Our study evaluated the effect of corticosteroid therapy on physiological myocardial FDG uptake in patients with CS and explored the relationship between FPG, FFA, and myocardial FDG uptake. The key findings of this study include a significant increase in physiological myocardial FDG uptake after corticosteroid therapy, as well as the identification of FFA as a potential predictor of physiological uptake, with higher sensitivity and specificity compared to FPG.

The frequency of physiological myocardial FDG uptake was significantly higher after the initiation of corticosteroid therapy compared to the pre-treatment phase. While FDG PET has been recognized as a valuable diagnostic tool for assessing myocardial inflammation in CS [12–14], the increased frequency of physiological FDG uptake after corticosteroid therapy introduces a potential confounder. This issue not only affects visual assessments but also the use of quantitative PET metrics, such as SUVmax, cardiac metabolic volume, and cardiac metabolic activity, which are essential for quantifying inflammatory burden and monitoring treatment response [3, 15]. If physiological uptake is misinterpreted as residual inflammation, it may lead to overtreatment or misjudgment of disease activity, underlining the need for careful interpretation of post-treatment FDG PET images, especially when suppression of physiological uptake is incomplete.

The increased physiological uptake post-therapy is likely related to the metabolic effects of corticosteroids. Although glucocorticoids acutely promote lipolysis and elevate circulating FFA levels, chronic exposure paradoxically reduces plasma FFA concentrations. This effect is primarily driven by glucocorticoid-induced hyperinsulinemia, which suppresses hormone-sensitive lipase activity and enhances hepatic re-esterification of FFAs into triglycerides, favoring lipid storage [9]. This biphasic metabolic response helps explain the observed FFA reduction in our cohort despite prolonged fasting, and supports the hypothesis that steroid-induced metabolic shifts underlie the increased myocardial glucose uptake and physiological FDG accumulation.

Furthermore, myocardial FFA metabolism is influenced not only by circulating FFA levels but also by intracellular transport and mitochondrial β-oxidation. Although direct evidence in cardiomyocytes is limited, systemic corticosteroid-induced metabolic changes may reduce myocardial FFA availability and shift cardiac energy metabolism toward glucose dependence. As a result, even with adequate dietary preparation, physiological FDG uptake may increase due to enhanced myocardial glucose utilization. Under normal fasting conditions, the myocardium predominantly utilizes FFAs as its primary energy substrate, with minimal reliance on glucose metabolism [16]. The decrease in FFA levels observed in our study suggests that corticosteroid therapy alters this balance, promoting glucose dependence and contributing to increased physiological FDG uptake. This observation is consistent with prior reports that emphasize the suppressive role of elevated FFA levels on physiological FDG accumulation in the heart [5]. Our ROC analysis further supports this interpretation, demonstrating that an FFA threshold of 615 µEq/L was highly predictive of physiological uptake, with a sensitivity of 100% and specificity of 83.6%. In contrast, FPG failed to reliably differentiate physiological from pathological uptake patterns, further validating the superior utility of FFA as a preparatory marker for cardiac FDG PET. Therefore, measuring FFA levels before FDG PET acquisition may improve scan interpretability and reduce false-positive results due to physiological uptake. However, in routine clinical practice, it is not always feasible to measure plasma FFA immediately before imaging or to modify the examination protocol based on its value. Therefore, FFA should be considered primarily as a reference parameter to support the interpretation of myocardial FDG uptake, rather than as a determinant for altering the imaging protocol. Importantly, the observed changes in myocardial FDG uptake are unlikely to be explained solely by temporal factors. Although the interval between pre- and post-treatment scans may introduce potential confounding effects, our findings suggest a predominant influence of corticosteroid-related metabolic changes. In this retrospective study, detailed information regarding the exact interval from initiation of corticosteroid therapy to the post-treatment FDG PET scan was not consistently available. However, at the time of imaging, patients were generally categorized into distinct clinical phases, including periods of higher and lower corticosteroid exposure, rather than a uniform tapering process.

In addition, subgroup analysis including patients undergoing lower-dose corticosteroid therapy showed metabolic profiles comparable to pre-treatment conditions, with a low frequency of physiological myocardial FDG uptake. Taken together, these findings support the interpretation that the observed changes in myocardial FDG uptake are more consistent with dose-dependent metabolic effects of corticosteroids rather than the passage of time alone.

However, myocardial FDG uptake is determined by multiple physiological and pathological factors and should not be interpreted solely based on plasma FFA or FPG levels. In addition to substrate availability, myocardial glucose utilization is influenced by insulin levels, duration of fasting, and neurohumoral factors. Furthermore, underlying myocardial conditions such as active inflammation, myocardial injury, or perfusion abnormalities may also affect FDG uptake independently of metabolic preparation. In this context, although our findings suggest an association between FFA levels and suppression of physiological myocardial FDG uptake, this relationship should be interpreted within the broader framework of myocardial metabolic regulation. The observed FDG uptake patterns likely reflect a complex interplay between metabolic preparation and disease-related factors.

Accordingly, FFA should be interpreted as a surrogate marker of the underlying metabolic state rather than a sole determinant of myocardial FDG uptake. The applicability of the proposed FFA threshold to FDG PET performed before the initiation of corticosteroid therapy remains uncertain. Because corticosteroid administration alters systemic metabolic conditions, including substrate utilization and hormonal regulation, the metabolic environment in the pre-treatment setting differs from that during therapy. Therefore, the present findings should not be directly extrapolated to the initial diagnostic evaluation of CS.

In contrast, during maintenance-dose corticosteroid therapy, metabolic conditions appeared to be closer to the pre-treatment state. In our exploratory analysis, plasma FFA and fasting glucose levels during lower-dose corticosteroid therapy were not significantly different from pre-treatment values, and physiological myocardial FDG uptake was infrequent. These findings suggest that suppression of physiological uptake may be more readily achieved during maintenance therapy. To improve clinical interpretability, we additionally performed an analysis including patients receiving maintenance-dose corticosteroid therapy. This analysis demonstrated that metabolic parameters during maintenance therapy were comparable to pre-treatment levels, and physiological myocardial FDG uptake was infrequent. These findings provide additional context for interpreting FDG PET findings across different phases of corticosteroid therapy. However, because of the limited number of cases with physiological uptake in this subgroup, the applicability of an FFA-based cutoff for distinguishing physiological from pathological uptake in the maintenance setting could not be reliably established and requires further investigation. Overall, the present findings are most directly supported in the context of higher corticosteroid exposure during the treatment phase. Although exploratory analyses suggest that similar metabolic relationships may be present during lower-dose therapy, the limited number of cases precludes definitive conclusions. Therefore, the results should be interpreted with consideration of the treatment phase.

Patients with CS often require immediate initiation of corticosteroid therapy due to the risk of life-threatening complications, including arrhythmias, conduction abnormalities, and sudden cardiac death [17, 18]. Early treatment, particularly when started before the development of left ventricular dysfunction, has been associated with improved clinical outcomes [19]. Systemic corticosteroids at moderate to high doses are widely accepted as the standard initial therapy for CS, due to their effectiveness in suppressing myocardial inflammation and stabilizing cardiac function. Furthermore, corticosteroid responsiveness is integrated into the diagnostic criteria established by the Heart Rhythm Society [12]. In this context, FDG PET plays a critical role not only in diagnosing CS but also in guiding treatment decisions and monitoring therapeutic response. The relatively high frequency of physiological myocardial FDG uptake observed during periods of higher corticosteroid exposure may represent a potential limitation in the evaluation of treatment response. In the present study, reduced plasma FFA levels and increased glucose utilization during higher corticosteroid exposure may promote physiological FDG uptake, thereby complicating interpretation of residual inflammatory activity. In contrast, during lower-dose corticosteroid therapy, metabolic conditions were closer to the pre-treatment state, and physiological uptake was infrequent. These findings suggest that FDG PET interpretation during higher corticosteroid exposure may be more susceptible to metabolic confounding and should be performed with particular caution. However, our findings suggest that corticosteroid therapy may complicate the interpretation of post-treatment FDG PET scans by increasing physiological myocardial uptake, especially when FFA suppression is inadequate. These findings highlight the need for careful evaluation of FDG PET results in CS patients undergoing corticosteroid therapy, with consideration of metabolic factors that may influence scan interpretation.

This study has several limitations. This was a retrospective, single-center analysis with a relatively small sample size. Selection bias may have influenced the results, as all included patients had undergone corticosteroid therapy, and thus the findings may not represent the full clinical spectrum of CS. FFA measurements were not available in all patients because FFA is not routinely assessed in clinical practice and its availability depended on institutional and logistical factors. In addition, availability of FFA measurement changed during the study period due to institutional and logistical factors. Importantly, missing data occurred at different time points, with some patients having FFA values only at baseline and others only at follow-up. This pattern suggests that missingness was not restricted to specific patient groups. Although this may introduce potential selection bias, no significant differences were observed between patients with and without FFA data, and the impact on the main findings is likely to be limited. The preparation protocol was not entirely uniform due to the retrospective nature of the study. Although patients were instructed to follow a low-carbohydrate diet with prolonged fasting, variations in dietary adherence and institutional practice occurred during the study period. In particular, unfractionated heparin was used in earlier cases but was discontinued in later cases. However, based on our previous findings, prolonged fasting combined with a low-carbohydrate diet is the primary determinant of suppression of physiological myocardial FDG uptake, whereas unfractionated heparin has only a minimal additional effect. Therefore, these variations are unlikely to have substantially influenced the main findings. Myocardial perfusion imaging was not routinely performed in conjunction with FDG PET. Although combined perfusion and metabolic imaging is often used to improve diagnostic confidence, all subjects had already fulfilled established clinical diagnostic criteria for cardiac sarcoidosis prior to inclusion. Furthermore, this study focused on longitudinal changes in FDG uptake patterns in a treatment setting rather than diagnostic evaluation. Therefore, while the absence of perfusion imaging may represent a limitation, its impact on the primary findings of this study is expected to be limited, given the focus on longitudinal changes in FDG uptake patterns within the same patients. Finally, histologic confirmation of myocardial involvement was not available in all cases, which may limit the correlation between imaging findings and pathological inflammation.

In conclusion, corticosteroid therapy in patients with CS is associated with an increased frequency of physiological myocardial FDG uptake, likely reflecting dose-dependent metabolic alterations, including reduced plasma FFA levels and enhanced myocardial glucose utilization. While plasma FFA may serve as a useful surrogate marker of the underlying metabolic state, myocardial FDG uptake is influenced by multiple physiological and pathological factors and should not be interpreted solely based on FFA levels.

## Electronic Supplementary Material

Below is the link to the electronic supplementary material.

Supplementary Material 1

## Data Availability

The datasets used and/or analyzed in the current study are available from the corresponding author upon request.

## References

[1] Drent M, Crouser ED, Grunewald J. Challenges of Sarcoidosis and Its Management. N Engl J Med. 2021;385(11):1018–32. 10.1056/NEJMra2101555. 34496176 10.1056/NEJMra2101555

[2] Lynch JP 3rd, Hwang J, Bradfield J, Fishbein M, Shivkumar K, Tung R. Cardiac involvement in sarcoidosis: evolving concepts in diagnosis and treatment. Semin Respir Crit Care Med. 2014;35(3):372–90. 10.1055/s-0034-1376889. 25007089 10.1055/s-0034-1376889 PMC4253029

[3] Osborne MT, Hulten EA, Singh A, Waller AH, Bittencourt MS, Stewart GC, Hainer J, Murthy VL, Skali H, Dorbala S, Di Carli MF, Blankstein R. Reduction in ¹⁸F-fluorodeoxyglucose uptake on serial cardiac positron emission tomography is associated with improved left ventricular ejection fraction in patients with cardiac sarcoidosis. J Nucl Cardiol. 2014;21(1):166–74. 10.1007/s12350-013-9828-6. 24307261 10.1007/s12350-013-9828-6

[4] Oyama-Manabe N, Manabe O, Aikawa T, Sobue Y, Asakura R. Updates on Cardiac MRI and PET Imaging for the Diagnosis and Monitoring of Cardiac Sarcoidosis. Korean J Radiol. 2025;26(9):804–16. 10.3348/kjr.2025.0148. 40873372 10.3348/kjr.2025.0148 PMC12394825

[5] Manabe O, Yoshinaga K, Ohira H, Masuda A, Sato T, Tsujino I, Yamada A, Oyama-Manabe N, Hirata K, Nishimura M, Tamaki N. The effects of 18-h fasting with low-carbohydrate diet preparation on suppressed physiological myocardial (18)F-fluorodeoxyglucose (FDG) uptake and possible minimal effects of unfractionated heparin use in patients with suspected cardiac involvement sarcoidosis. J Nucl Cardiol. 2016;23(2):244–52. 10.1007/s12350-015-0226-0. 26243179 10.1007/s12350-015-0226-0 PMC4785205

[6] Ohira H, Tsujino I, Yoshinaga K. (1)(8)F-Fluoro-2-deoxyglucose positron emission tomography in cardiac sarcoidosis. Eur J Nucl Med Mol Imaging. 2011;38(9):1773–83. 10.1007/s00259-011-1832-y. 21559980 10.1007/s00259-011-1832-y

[7] Ohira H, Birnie DH, Pena E, Bernick J, Mc Ardle B, Leung E, Wells GA, Yoshinaga K, Tsujino I, Sato T, Manabe O, Oyama-Manabe N, Nishimura M, Tamaki N, Dick A, Dennie C, Klein R, Renaud J, deKemp RA, Ruddy TD, Chow BJ, Davies R, Hessian R, Liu P, Beanlands RS, Nery PB. Comparison of (18)F-fluorodeoxyglucose positron emission tomography (FDG PET) and cardiac magnetic resonance (CMR) in corticosteroid-naive patients with conduction system disease due to cardiac sarcoidosis. Eur J Nucl Med Mol Imaging. 2016;43(2):259–69. 10.1007/s00259-015-3181-8. 26359191 10.1007/s00259-015-3181-8

[8] Rojulpote C, Bhattaru A, Patil S, Adams SL, Salas JA, Vidula MK, Perez RP, Kc W, Patterson K, Clancy CB, Rossman M, Goldberg L, Bravo PE. Assessing the effect of repeat positron emission tomography imaging on treatment response and cardiovascular outcomes among a homogenously treated cohort of patients with suspected cardiac sarcoidosis. J Nucl Cardiol. 2025;43:102082. 10.1016/j.nuclcard.2024.102082. 39579922 10.1016/j.nuclcard.2024.102082

[9] Macfarlane DP, Forbes S, Walker BR. Glucocorticoids and fatty acid metabolism in humans: fuelling fat redistribution in the metabolic syndrome. J Endocrinol. 2008;197(2):189–204. 10.1677/JOE-08-0054. 18434349 10.1677/JOE-08-0054

[10] Terasaki F, Azuma A, Anzai T, Ishizaka N, Ishida Y, Isobe M, Inomata T, Ishibashi-Ueda H, Eishi Y, Kitakaze M, Kusano K, Sakata Y, Shijubo N, Tsuchida A, Tsutsui H, Nakajima T, Nakatani S, Horii T, Yazaki Y, Yamaguchi E, Yamaguchi T, Ide T, Okamura H, Kato Y, Goya M, Sakakibara M, Soejima K, Nagai T, Nakamura H, Noda T, Hasegawa T, Morita H, Ohe T, Kihara Y, Saito Y, Sugiyama Y, Morimoto SI, Yamashina A, Group JCSJW. JCS 2016 Guideline on Diagnosis and Treatment of Cardiac Sarcoidosis - Digest Version. Circ J. 2019;83(11):2329–88. 10.1253/circj.CJ-19-0508. 31597819 10.1253/circj.CJ-19-0508

[11] Ohira H, Ardle BM, deKemp RA, Nery P, Juneau D, Renaud JM, Klein R, Clarkin O, MacDonald K, Leung E, Nair G, Beanlands R, Birnie D. Inter- and Intraobserver Agreement of (18)F-FDG PET/CT Image Interpretation in Patients Referred for Assessment of Cardiac Sarcoidosis. J Nucl Med. 2017;58(8):1324–9. 10.2967/jnumed.116.187203. 28254873 10.2967/jnumed.116.187203

[12] Birnie DH, Sauer WH, Bogun F, Cooper JM, Culver DA, Duvernoy CS, Judson MA, Kron J, Mehta D, Cosedis Nielsen J, Patel AR, Ohe T, Raatikainen P, Soejima K. HRS expert consensus statement on the diagnosis and management of arrhythmias associated with cardiac sarcoidosis. Heart rhythm: official J Heart Rhythm Soc. 2014;11(7):1305–23. 10.1016/j.hrthm.2014.03.043. 10.1016/j.hrthm.2014.03.043 24819193

[13] Aggarwal NR, Snipelisky D, Young PM, Gersh BJ, Cooper LT, Chareonthaitawee P. Advances in imaging for diagnosis and management of cardiac sarcoidosis. Eur Heart J Cardiovasc Imaging. 2015;16(9):949–58. 10.1093/ehjci/jev142. 26104960 10.1093/ehjci/jev142

[14] Tamaki N, Manabe O. Current status and perspectives of nuclear cardiology. Ann Nucl Med. 2024;38(1):20–30. 10.1007/s12149-023-01878-1. 37891375 10.1007/s12149-023-01878-1

[15] Ahmadian A, Brogan A, Berman J, Sverdlov AL, Mercier G, Mazzini M, Govender P, Ruberg FL, Miller EJ. Quantitative interpretation of FDG PET/CT with myocardial perfusion imaging increases diagnostic information in the evaluation of cardiac sarcoidosis. J nuclear cardiology: official publication Am Soc Nuclear Cardiol. 2014;21(5):925–39. 10.1007/s12350-014-9901-9. 10.1007/s12350-014-9901-9 24879453

[16] Lopaschuk GD, Ussher JR, Folmes CD, Jaswal JS, Stanley WC. Myocardial fatty acid metabolism in health and disease. Physiol Rev. 2010;90(1):207–58. 10.1152/physrev.00015.2009. 20086077 10.1152/physrev.00015.2009

[17] Ayyala US, Nair AP, Padilla ML. Cardiac sarcoidosis. Clinics in chest medicine. 2008;29(3):493–508, ix. 10.1016/j.ccm.2008.03.005 10.1016/j.ccm.2008.03.005 18539240

[18] Kim JS, Judson MA, Donnino R, Gold M, Cooper LT Jr., Prystowsky EN, Prystowsky S. Cardiac sarcoidosis. Am Heart J. 2009;157(1):9–21. 10.1016/j.ahj.2008.09.009. 19081391 10.1016/j.ahj.2008.09.009

[19] Yazaki Y, Isobe M, Hiroe M, Morimoto S, Hiramitsu S, Nakano T, Izumi T, Sekiguchi M. Central Japan Heart Study G. Prognostic determinants of long-term survival in Japanese patients with cardiac sarcoidosis treated with prednisone. Am J Cardiol. 2001;88(9):1006–10. 11703997 10.1016/s0002-9149(01)01978-6

