# Supplementary material for: Interpretation of myocardial FDG uptake during corticosteroid therapy in cardiac sarcoidosis evaluation with 18F-FDG PET/CT
Source: Ann Nucl Med. 2026 May 11;40(9):1053–62. doi: 10.1007/s12149-026-02222-z (PMC13506651; doi:10.1007/s12149-026-02222-z)
Supplement: Supplementary file 1 [file 12149_2026_2222_MOESM1_ESM.docx]

Supplementary Figure. Representative patterns of myocardial FDG uptake

Representative whole-body maximum intensity projection (MIP) images (upper row) and corresponding axial fused PET/CT images (lower row) demonstrating the classification of myocardial FDG uptake patterns.

Myocardial uptake was categorized into five patterns based on visual assessment:

None: Complete suppression of myocardial FDG uptake.

Diffuse: Homogeneous and diffuse uptake involving the entire left ventricular myocardium.

Focal: Discrete focal uptake in one or more myocardial segments.

Focal-on-diffuse: Focal areas of increased uptake superimposed on a background of diffuse myocardial uptake.

Isolated basal/lateral uptake: Localized uptake predominantly involving the basal and/or lateral wall, without diffuse background activity.
